# Microbial interaction-induced siderophore dynamics lead to phenotypic differentiation of *Staphylococcus aureus*

**DOI:** 10.3389/fcimb.2023.1277176

**Published:** 2023-11-17

**Authors:** Soundarya Rajapitamahuni, Eun Sun Lyou, Bo Ram Kang, Tae Kwon Lee

**Affiliations:** Department of Environmental and Energy Engineering, Yonsei University, Wonju, Republic of Korea

**Keywords:** siderophores, microbial interactions, iron deficiency, phenotype, Raman spectroscopy

## Abstract

This study investigated the impact of microbial interactions on siderophore dynamics and phenotypic differentiation of *Staphylococcus aureus* under iron-deficient conditions. Optimization of media demonstrated that the glycerol alanine salts medium was best suited for analyzing the dynamics of siderophore production because of its stable production of diverse siderophore types. The effects of pH and iron concentration on siderophore yield revealed a maximum yield at neutral pH and low iron concentration (10 µg). Microbial interaction studies have highlighted variations in siderophore production when different strains (*Staphylococcus epidermidis*, *Pseudomonas aeruginosa*, and *Escherichia coli*) are co-cultured with *S. aureus*. Co-culture of *S. aureus* with *P. aeruginosa* eliminated siderophore production in *S. aureus*, while co-culture of *S. aureus* with *E. coli* and *S. epidermidis* produced one or two siderophores, respectively. Raman spectroscopy revealed that microbial interactions and siderophore dynamics play a crucial role in directing the phenotypic differentiation of *S. aureus*, especially under iron-deficient conditions. Our results suggest that microbial interactions profoundly influence siderophore dynamics and phenotypic differentiation and that the study of these interactions could provide valuable insights for understanding microbial survival strategies in iron-limited environments.

## 1 Introduction

Iron is an indispensable micronutrient required for the growth and survival of microorganisms (Murdoch and Skaar, 2022). Its bioavailability in nature is often limited, which leads to competitive dynamics among microbial species. Siderophores are small, iron-chelating molecules secreted by microorganisms that play a critical role in this competition (Perraud et al., 2022). By scavenging and transporting iron, siderophores enhance the bioavailability of iron, thus allowing the microbes in which they are present to thrive or outcompete other microorganisms in iron-scarce environments (Kramer et al., 2020). They also exhibit antimicrobial properties by depriving pathogenic microbes of iron, thereby inhibiting their growth (Ribeiro and Simões, 2019). To survive in competition with coexisting microorganisms in nature, microorganisms produce their own siderophores to gain a competitive advantage (Hibbing et al., 2010). Thus, research on siderophore dynamics is crucial for understanding microbial interactions, as it offers insights into competition among microbes and their survival strategies in iron-limited environments.

The type of siderophore produced by bacteria varies depending on the surrounding environment, particularly in response to coexisting bacterial species (Leventhal et al., 2019). Among diverse microbial communities, this adaptation allows bacteria to produce siderophores with greater specificity and affinity for iron, thereby increasing their competitive advantage in acquiring this essential nutrient. Microorganisms have evolved to produce siderophores that possess different chemical groups to outcompete other microorganisms for iron (Shepherdson et al., 2022). For example, enterobactin, pyochelin, hydroxamatelcaligin, and xanthoferrin are species-specific siderophores that contain catecholate, phenolate, hydroxamate, and carboxylate groups, respectively (Ahmed and Holmström, 2014). The ability to produce siderophores with greater specificity and affinity for iron provides such bacteria to have a competitive edge over other microbial communities. The variability in siderophore production, both in terms of specificity and chemical groups, showcases a strategic survival mechanism and interplay among bacterial communities. This fact highlights siderophores as potential markers for studying microbial interactions, community dynamics, and evolution of competitive strategies. Furthermore, the observation that specific siderophores are linked to a particular species could provide a new perspective on distinguishing microbial species and studying their interactions based on their unique siderophore profiles.

*Staphylococcus aureus*, an opportunistic human pathogen, is the leading cause of bacterial infections in the anterior nares and skin of healthy individuals (Kwiecinski and Horswill, 2020). *S. aureus* is uniquely known to produce siderophores with different chemical structures, including staphyloferrin A, staphyloferrin B, staphylobactin, and aureochelin (Ghssein and Ezzeddine, 2022). The structures of staphyloferrin A and B are well known for their polycarboxylate-type siderophores (Zhang et al., 2020), and staphyloferrin B is known to repress the production of staphyloferrin A in the same medium (Sheldon et al., 2014). In contrast, phenolate-catecholate siderophores such as aureochelins from *S. aureus* have long been reported to be produced in a different chemically defined medium (CDM) (Courcol et al., 1997), implying that there are specific precursors that support the biosynthesis of various siderophore structures in a specific environment. However, the effect of interspecies interactions on siderophore production in *S. aureus* remains largely unexplored. Understanding the mechanisms by which the presence of other bacterial species affects siderophore production in *S. aureus* will provide valuable insights into the dynamics of siderophore production.

In this study, we aimed to optimize the composition of a growth medium to maximize the production of all three siderophores (staphyloferrin A, staphyloferrin B, and aureochelin) by *S. aureus* and to investigate how the dynamics of siderophore production are influenced by co-culturing different microbial species with each other. To focus on chemical communication between species, we co-cultured *P. aeruginosa*, *S. epidermidis*, and *E. coli* with *S. aureus* using transwell plates with 0.4 mm membranes under iron-deficient conditions. To achieve this, we used the Glycerol alanine salts (GAST) medium to create iron-limited conditions and conducted a series of co-culture experiments to analyze siderophore production over time. In addition, cell viability was examined by flow cytometry to determine whether co-culture inhibited the growth of *S. aureus*. Raman spectroscopy analysis of single cells of *S. aureus* was performed to track phenotypic changes in *S. aureus*. Our findings provide new insights into the role of interspecies interactions of bacteria in shaping siderophore production by *S. aureus* and highlight the importance of understanding the factors governing the production and function of these vital iron-scavenging molecules in microbial communities.

## 2 Materials and methods

### 2.1 Experimental design

The experiments were designed to characterize the effect of co-cultured bacteria on siderophore production by *S. aureus*, specifically focusing on communication among them. To study microbial interaction, a co-culture system was established utilizing a 24 mm-transwell inserts plate with a permeable membrane (0.4 µm) that are designed as two compartments which helps in the passage of metabolites without any physical interaction. The culture media for both axenic and co-culture conditions were prepared identically, except for the iron concentration, which served as the sole variable in each sample. *Staphylococcus aureus* NCTC 8325-4 (designated as SA) was cultured in both axenic conditions or co-cultivated with *Staphylococcus epidermidis* ATCC 12228 (SE), *Pseudomonas aeruginosa* KCTC 2004 (PA), and *Escherichia coli* KCTC 1116 (EC). The cultures were grown in media containing sufficient iron concentration or was deficient in iron content. After 24 and 48 h, optical density at 600 nm (OD600) was measured, and characterization of siderophores, ROS levels, flow cytometry, and Raman spectroscopy analysis were evaluated to observe the impact of co-culture on siderophore production in *S. aureus* and its overall biochemical phenotype.

### 2.2 Optimization of media, carbon sources, iron concentration, and pH for siderophore production

SA was cultured in three different iron-deficient media such as GAST, Fiss minimal media (FISS), and Chemically Defined Low Iron media (CDLIM) (De Voss et al., 2000; Dimopoulou et al., 2021). Subsequently, several physicochemical parameters such as pH, carbon source, and iron concentration were investigated to optimize siderophore production. Cell growth and siderophore yield were assessed every 12 h. In the GAST medium, glycerol was substituted with 20.0 g/l of lactose, galactose, maltose, xylose, or glucose as the carbon source. The pH was adjusted from 4 to 10. To determine the iron concentration threshold that represses siderophore biosynthesis in SA, the culture was grown in GAST medium externally supplemented with 0 to 300 μg of iron (FeCl_3_·6H_2_0). All culture flasks were incubated in a rotary shaker at 27°C at 150 rpm. Cell growth was measured using a spectrophotometer at OD600.

The selected GAST medium for the growth experiments had the following composition (per liter):0.3 g Bacto Casitone (Difco Becton, Becton, NJ, USA), 0.05 g ferric ammonium citrate, 4.0 g dibasic potassium phosphate, 2.0 g citric acid, 1.0 g l-alanine, 1.2 g magnesium chloride hexahydrate, 0.6 g potassium sulfate, 2.0 g ammonium chloride, 1.80 ml 10 N sodium hydroxide, and 10.0 ml glycerol. To achieve iron-deficient conditions, ferric ammonium citrate was omitted in the medium. After addition of all the components, the medium was filter-sterilized using a 0.2 μm membrane filter. Bacterial strains were first grown in Tryptic Soy Broth (TSB) overnight at 34°C, and these preculture strains were centrifuged to obtain pellets. Next, starting with an OD_600_ of 0.05, the cell pellets were simultaneously added to the respective iron-limited medium.

### 2.3 Deferration of glassware and media

All glassware employed in this study were thoroughly cleaned using 6 N HCl to remove residual iron, rinsed multiple times with deionized water, and subsequently oven-dried. To further eliminate iron from the chemicals used for siderophore production, an 8-hydroxyquinoline chloroform solution (Sigma-Aldrich, St. Louis, MO, USA) was used. Chloroform was added to the solution, which was vigorously shaken to promote the formation of two distinct layers. The bottom chloroform layer was carefully separated, and this separation process was repeated 3 to 4 times to ensure complete removal of iron from the chemicals.

### 2.4 The chrome azurol S assay for siderophore production

Siderophore production by the bacteria was assessed using the universal CAS assay (Schwyn and Neilands, 1987). Siderophore production is strain- and media-dependent (Schwyn and Neilands, 1987). Each bacterial strain was grown aerobically in various iron-limiting media, specifically designed and optimized for siderophore production, at 28°C at 120 rpm for 120 h. Siderophores are extracellular products found in culture supernatants. The broth culture was centrifuged at 9,000 rpm for 10 min at 4°C, and supernatants were collected and cell pellets discarded.

During the initial selection of media, the broth was harvested at 24, 48, 72, 96, and 120 h, and the obtained supernatants were passed through 0.2 µm filters [PES (polyethersulfone); Hyundai Micro, Republic of Korea] to remove residual cell debris. The supernatant was then centrifuged at 9,000 rpm for 10 min, and the cell-free supernatant was analyzed using the CAS assay. Siderophore production was assessed at 630 nm, and the relative siderophore content was calculated using the following formula and expressed as percent siderophore units (% SU):


$$\%\;SU=\frac{Ar-As}{Ar}*100$$

where Ar is the absorbance of the reference (deionized water), and As is the absorbance of the sample (culture supernatant) at 630 nm. Deferoxamine mesylate (Sigma-Aldrich, St. Louis, MO, USA), a hydroxamate siderophore, was used as a standard for the CAS assay (Calvente et al., 2001).

To increase the iron exchange rate during the CAS assay of different siderophore types, 10 µL of 5-sulfosalicylic acids as a shuttle solution was added. Typically, in a CAS assay, a positive result is indicated by color change from blue to orange/pinkish red within a few minutes. However, some siderophores such as α- hydroxycarboxylic acid require hours, while rhodotorulic acid needs days to elicit a color change. This indicates that the iron complexes formed during the reaction are stable. Therefore, it is crucial to consider and analyze the reaction rate involved in the change in color for analytical purposes (Murakami et al., 2021).

### 2.5 Chemical characterization of siderophores

The CAS assay was employed to measure the total yield of siderophores, irrespective of their structures. The following methods were used to measure the yield of siderophores with three different structures.

To detect hydroxamate-type siderophores, an equal amount of culture supernatant was mixed with 6 N H_2_SO_4_ and hydrolyzed in a boiling water bath for 6 h. Next, 3 ml Na-acetate (for buffering), 1 ml sulfanilic acid, and 0.5 ml iodine solution were added to the solution. After 5 min, 1 ml of Na-arsenite and 1 ml of alpha-naphthylamine were added to remove iodine residues from the solution. The entire content was diluted in 10 ml distilled water, and a change in the color of the solution was observed after 20–30 min of incubation. Absorbance of the reaction mixture was measured at 526 nm. This method is known as the Csaky assay (Csáky et al., 1948); deferoxamine methanesulfonate salt was used as a standard.

Catecholates were determined by adding equal proportions of culture supernatant to a mixture of 5 N HCl, 0.5 ml nitrite-molybdate (color changes to yellow) and 10 N NaOH (color changes to red, stable for 1 h). The absorbance was measured at 510 nm using a spectrophotometer Spark (Tecan, Männedorf, Switzerland). 2, 3-dihydroxybenzoic acid was used as a standard (Arnow, 1937).

Carboxylate activity was detected by adding 1 mL culture supernatant to 250 µl of 0.25 mM CuSO_4_ and 2 ml of acetate buffer (pH 4). The presence of a carboxylate group in the siderophore was indicated by a color change from yellow to pink. The absorbance spectra of the solutions were measured at 190-280 nm, and a positive result was indicated by a peak in this range (Osman et al., 2019).

### 2.6 Growth of axenic and co-cultured strains in iron-deficient and iron-rich conditions

We used two different conditions, iron-deficient and iron-rich, for both axenic and co-culture samples. All bacterial cultures were diluted, and the initial OD600 was set to 0.05 in GAST medium. To obtain axenic cultures, the same species of bacteria (1.5 ml) were loaded in both transwell insert and plate. In the co-cultures, SA (1.5 ml) was loaded in the plate, whereas SE, PA, and EC (1.5 ml) were loaded in the Transwell insert. The transwell insert plates were incubated at 34°C for 48 h and gently shaken (45 rpm) to avoid diffusion of metabolites between the compartments. The samples were analyzed by determining OD_600_, Raman spectra, and flow cytometric features at 24 and 48 h.

### 2.7 Flow cytometry

To examine the viability of SA during co-culture with other bacterial species, we measured the viability of SA grown in both axenic and co-cultures for 24 and 48 h. We used the LIVE/DEAD™ BacLight™ bacterial viability and counting kit (Invitrogen, Carlsbad, CA, USA),. Staining was performed according to manufacturer’s instructions. Initially, all samples were diluted 1:100 in phosphate-buffered saline and stained with 0.15 vol% 3.34 mM SYTO 9 for total cell analysis and with 0.15 vol% of 30 mM propidium iodide for live/dead cell analysis. The samples were analyzed using a CytoFLEX V0-B3-R2 flow cytometer (Beckman Coulter, CA, USA) equipped with two scatter detectors, five fluorescence detectors, and two lasers (blue 488 nm laser and red 638 nm laser). Fluorescence was collected in the green (FITC-A/FL1) and red (PE-A/FL2) channels, which are filters used to detect viable cells. Populations of live and dead bacterial cells were gated using a single stain containing SYTO9 and propidium iodide. A minimum of 30,000 cells were collected per sample.

### 2.8 ROS determination

#### 2.8.1 H_2_O_2_ measurement

Bacterial cells were harvested by centrifugation (9,000 rpm, 15 min, 4°C), and the cell pellet was resuspended in 0.1% w/v trichloroacetic acid solution. The suspended culture was centrifuged again at 9,000 rpm for 10 min. An aliquot of 0.5 ml of the supernatant was mixed with 0.5 ml of 10 mM phosphate buffer (pH 7.5) and 1 ml of 1 M potassium iodide. Absorbance of the solution was measured at 390 nm (Velikova et al., 2000). H_2_O_2_ concentration (μM H_2_O_2_/g FW; Formula weight) in the sample was determined from a calibration curve prepared using known concentrations of H_2_O_2_.

#### 2.8.2 O_2_ measurement

Bacterial cells were harvested by centrifugation (9,000 rpm, 15 min, 4°C), resuspended in 5 ml of 65 mM potassium phosphate buffer (pH 7.5), and centrifuged again at 9,000 rpm for 5 min. An aliquot of 1 ml of the supernatant was mixed with 0.9 ml of 65 mM potassium phosphate buffer (pH 7.8) and 0.1 ml of 10 mM hydroxyl ammonium chloride. After incubation at 25°C for 20 min, 1 ml of 17 mM sulphanilic acid and 1 ml of 7 mM α-naphthylamine were added to the mixture. After further incubation for 20 min, absorbance of the solution was measured at 530 nm (Liu et al., 2010). Sodium nitrite was used as standard.

#### 2.8.3 OH measurement

Bacterial cells were harvested by centrifugation (9,000 rpm, 15 min, 4°C), resuspended in 2 ml of 50 mM potassium phosphate buffer (pH 7.0), and centrifuged again at 9,000 rpm for 5 min. Later, 0.5 ml of the supernatant thus obtained was mixed with 0.5 ml of 50 mM potassium phosphate buffer (pH 7.0) containing 2.5 mM of 2-deoxyribose. The reaction was carried out at 35°C in the dark for 1 h. After adding 1 ml of 1% TBA in 0.05 M sodium hydroxide and 1 ml of acetic acid, the mixture was boiled for 30 min and immediately cooled on ice. Absorbance of the solution was measured at 532 nm (Halliwell, 2006) and the OH content was expressed as absorbance units per gram of FW.

### 2.9 Raman spectroscopy

To investigate the effect of co-cultivation on phenotypic variance of SA, we collected Raman spectra ranging from 400 to 1800 cm^-1^ using used Raman spectroscopy. Initially, the bacterial cells were washed with PBS and centrifuged at 13,000 rpm for 5 minutes. Later, these samples were fixed with 1% formaldehyde in 4°C for 2 h, followed by washing twice with PBS buffer. Next, 1.5 µl bacterial cells were spotted on an aluminum-coated slide (LiMedion, Mannheim, Germany) and air dried at room temperature. Finally, the slides were washed with ultrapure water to remove saline particles and air-dried. Raman spectra were obtained using the Confocal Raman imaging system XperRam35V (Nanobase, Seoul, Republic of Korea) equipped with 1,800 g/mm gating, 532 nm neodymium-yttrium aluminum garnet laser, LTGL-532RL (Leading Tech) under an MPLFLN 40×objective (Olympus). The laser power applied to a single cell was 2.0 mW with an integrated duration of 25 s for each cell. Resulting scattered light was collected using an Atik 428EX Color CCD Camera (Atik Cameras). Preprocessing of the Raman spectra was performed using the R package ‘ChemoSpec (v5.3.11)’ (2021 bryan) using a baseline correction (function: als), normalization (function: TotInt), and alignment. Raman spectrum analysis was based on discriminant analysis of principal components (DAPC) in the R package ‘adegenet’.

## 3 Results

### 3.1 Optimization of media for multiple siderophore production

**Figure 1:**
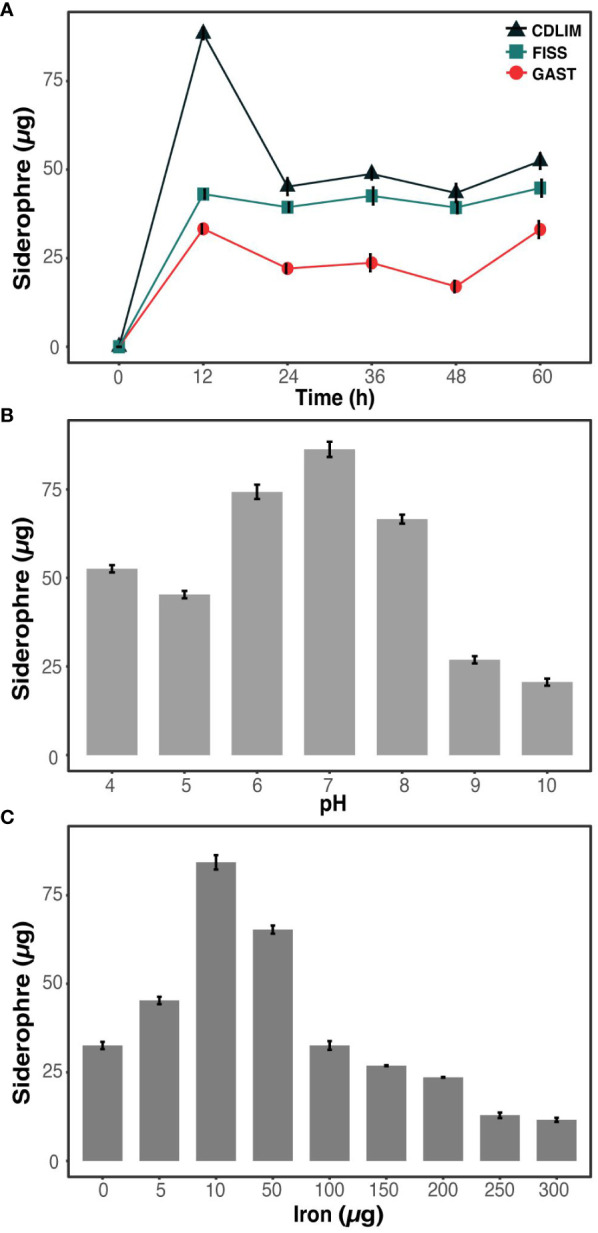
Optimization of media, pH, and iron concentration for enhanced siderophore production in *S. aureus* **(A)** Evaluation of media types (CDLIM, FISS, GAST) for optimal siderophore production in *S. aureus.* **(B)** Effects of pH optimization on siderophore production in *S. aureus* at 48 h. **(C)** Influence of iron concentration optimization on elevated siderophore production in *S. aureus* after 48 h incubation. Siderophores are quantitatively measured as µg/ml. The data represent mean± SD values of three replicates.

We compared three well-known media for siderophore production and selected the media-based yield, stability, diversity, and production rates of siderophores. In all the media, the concentration of siderophores increased rapidly during the first 12 h and tended to be either maintained (GAST) or decreased (FISS and CDLIM) after 12 h (**Figure 1A**). The average yields of siderophore produced from 24 h to 60 h were 48.9 ± 2.3, 45.2 ± 1.2, and 28.2 ± 1.4 µg for CDLIM, FISS, and GAST, respectively. Compared to CDLIM and FISS, GAST produced a uniform yield of siderophores over the incubation period. FISS and CDLIM temporarily produced both carboxylate- and catecholate-containing siderophores; however, for most of the incubation period, they specifically produced one type of siderophore (**Table 1**). GAST selectively produced only carboxylate-containing siderophores for the first 24 h, and later produced all three types of siderophores relatively evenly from 36 h onwards. Although GAST did not achieve the yield and production rate of siderophores compared to that of FISS, it was chosen as a suitable medium to study the dynamics of siderophore production by microbial interactions because it could stably produce three types of siderophores after 24 h.

**Table 1:** Production of different types of siderophores in three different media CDLIM, FISS, and GAST.

Time (h)	CDLIM			FISS			GAST		
	Catecholate (µg)	Carboxylate (µg)	Hydroxamate (µg)	Catecholate (µg)	Carboxylate (µg)	Hydroxamate (µg)	Catecholate (µg)	Carboxylate (µg)	Hydroxamate (µg)
**12**	–	36.2 ± 0.3	–	82.3 ± 0.4	32.6 ± 0.5	–	–	44.6 ± 1.5	–
**24**	–	24.6 ± 0.9	–	54.4 ± 1.4	–	–	19 ± 0.6	41.0 ± 0.4	–
**36**	–	28.1 ± 0.4	–	41.3 ± 0.7	–	–	25.4 ± 0.1	40.2 ± 1.2	33.2 ± 0.9
**48**	12.6 ± 0.3	30.5 ± 1.2	–	32.3 ± 0.2	–	–	29.2 ± 0.1	44.3 ± 0.8	36.5 ± 0.5
**60**	38.5 ± 0.3	–	–	44.5 ± 1.2		–	38.3 ± 0.4	51.6 ± 1.6	41.3 ± 0.7

Siderophores were quantified as µg/ml at different time periods. The data represent mean ± SD of three replicates.

The symbol "-" indicates that no such type of siderophore was produced.

The effects of pH and iron concentration on siderophore production are crucial. Iron solubility and bioavailability to growing microorganisms depend on the pH and iron concentration of a medium. We observed the highest siderophore yield (84.5 ± 2.3µg) at a pH of 7 (**Figure 1B**). As the pH deviated from the neutral pH (pH 7), either decreasing or increasing, siderophore production declined, reaching to 25 µg or lower. Changes in siderophore production were measured corresponding to the iron concentration (**Figure 1C**). When the iron concentration decreased from 300 to 10 µg, the siderophore production increased and reached its maximum yield (86 µg) at 10 µg iron concentration. For further experiments, the pH of the GAST medium was kept at a neutral pH and dose of iron at 10 µg.

### 3.2 Effect of microbial interaction on siderophore dynamics

The complex interplay between microbial cooperation and competition in a shared ecological niche shapes the dynamics of siderophore production through diverse strategies for survival and growth. We aimed to analyze the dynamics of siderophore production in SA by analyzing the differences in siderophores produced in co-cultures and monocultures. SE produced 42.0 ± 1.8 and 38.2 ± 1.2 µg of siderophores containing carboxylate and hydroxamate, respectively, when cultured as a monoculture (**Table 2**). PA produced a single type of siderophore, specifically hydroxamates (48.2 ± 0.2 µg), while EC produced only catecholate-type siderophores (42.5 ± 0.5 µg). In contrast, under co-culture conditions, some strains either ceased to produce siderophores or exhibited concentrations that were too low to be detected, potentially due to the presence of co-cultivating microorganisms. When SA was co-cultivated with PA, no siderophore production was observed in SA, while PA produced hydroxamate siderophore (33.1 ± 0.2 µg). Moreover, when SA was co-cultivated with SE, SA produced both carboxylate (54.3 ± 0.4 µg) and hydroxamate (19.2 ± 1.2 µg) siderophores, but not catecholate types, while SE produced only catecholate (32.4 ± 1.2 µg) siderophores. Interestingly, when SA was co-cultivated with EC, it produced a higher amount of carboxylate (61.1 ± 1.7 µg) along with a significant amount of catecholate (31.3 ± 0.2 µg) siderophores, whereas EC maintained its production solely of catecholate siderophores (53.1 ± 1.3 µg).

**Table 2:** Comparative analysis of siderophore types produced by *S. aureus* in axenic and co-culture conditions at 48* h*.

Conditions		Bacteria	Catechole (µg)	Carboxylate (µg)	Hydroxamate (µg)
**Axenic**		**SA**	29.2 ± 0.1	44.3 ± 0.8	36.5 ± 0.5
		**PA**	–	–	48 ± 0.2
		**SE**	–	42 ± 1.8	38 ± 1.2
		**EC**	42 ± 0.5	–	–
**Co-culture**	**SA+PA**	**SA**	–	–	–
		**PA**	–	–	33 ± 0.2
	**SA+SE**	**SA**	–	54 ± 0.3	19 ± 1.2
		**SE**	–	32 ± 0.2	–
	**SA+EC**	**SA**	31 ± 0.2	61 ± 1.6	–
		**EC**	53 ± 1.3	–	–

SA (axenic S. aureus), PA (axenic P. aeruginosa), SE (axenic S. epidermidis), EC (axenic E. coli), SA+PA (S. aureus co-cultured with P. aeruginosa), SA+SE (S. aureus co-cultured with S. epidermidis), SA+EC (S. aureus co-cultured with E. coli) Siderophores were quantitated as µg/ml at different time periods. The data represent mean± SD values of three replicates.

The symbol "-" indicates that no such type of siderophore was produced.

### 3.3 Effect of microbial interaction on growth and viability

**Figure 2:**
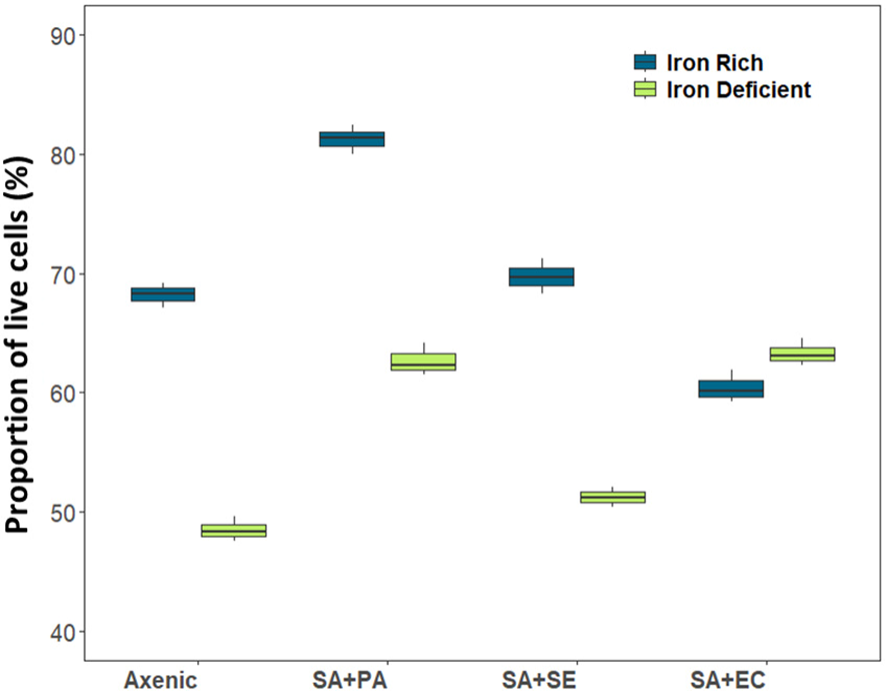
Viability of live cells in axenic and co-cultured *S. aureus* using flow cytometry in iron-rich and iron-deficient conditions at 48 h. X axis denotes axenic *S. aureus* (When *S. aureus* is present in both compartments) and *S. aureus* co-cultured/grown in transwell plates where in, one compartment contains *S. aureus* and other compartment contains other species. Axenic, axenic *S. aureus*; SA+PA: Measurement of viable cells of *S. aureus* when co-cultured with *P. aeruginosa* in another compartment, SA+SE: Measurement of viable cells of *S. aureus* when co-cultured with *S. epidermidis* in another compartment, SA+EC: Measurement of viable cells of *S. aureus* when co-cultured with *E. coli* in another compartment.

Differences between live and dead populations of SA was observed when co-cultured with SE, PA, or EC under both iron-rich and iron-deficient conditions (**Supplementary Figure 2**). The results revealed that under both iron-rich and iron-rich conditions, the growth of SA+PA significantly increased compared to that of both axenic and other co-cultured bacteria (**Supplementary Figure 1**). This was supported by flow cytometry analysis, wherein the viability of co-cultured (SA+PA) cells increased in comparison to the other co-cultured SA. A similar pattern was also observed in the axenic culture, which resembled the growth data. Gradually, the difference in viability between the axenic and co-cultured cells increased, and this difference was more pronounced with PA than with SE or EC. The variations in SA viability after co-culturing with SE and EC were comparable to the variations in axenic culture, indicating that SA was less influenced by co-culturing with these two strains. Although live cells in the iron-deficient media were significantly lower than those in iron-rich media, in both the cases, apart from co-cultivated SA+PA, all other co-cultivated bacteria exhibited similar viability after 48 h (**Figure 2**). We also observed that the number of dead cells was higher under iron-deficient conditions in all the cultures, except when co-cultured with PA (**Supplementary Figure 2**). Iron-sufficient and iron-deficient conditions were largely unaffected by the interactions, except for SA co-cultured with PA, although there was a minor increase in the growth of all co-cultures of SA grown in the iron-deficient medium.

Apart from the changes in the viability of SA, it was also important to observe the phenotypic changes in the microbial partners; hence, after observing the changes in SA, we also checked the changes in the growth of co-cultured partners (SE), (PA), and (EC) with their respective axenic cultures. Under iron-rich conditions, both SE and EC significantly increased when co-cultured, compared to axenic cultures, but in PA, the co-cultured cells decreased, and axenic culture showed the highest cell density when compared to all other cultures in both types. A similar trend was observed in iron-deficient conditions, where the co-cultures of SE and EC increased (**Supplementary Figures 3A, B**).

### 3.4 ROS generation

**Figure 3:**
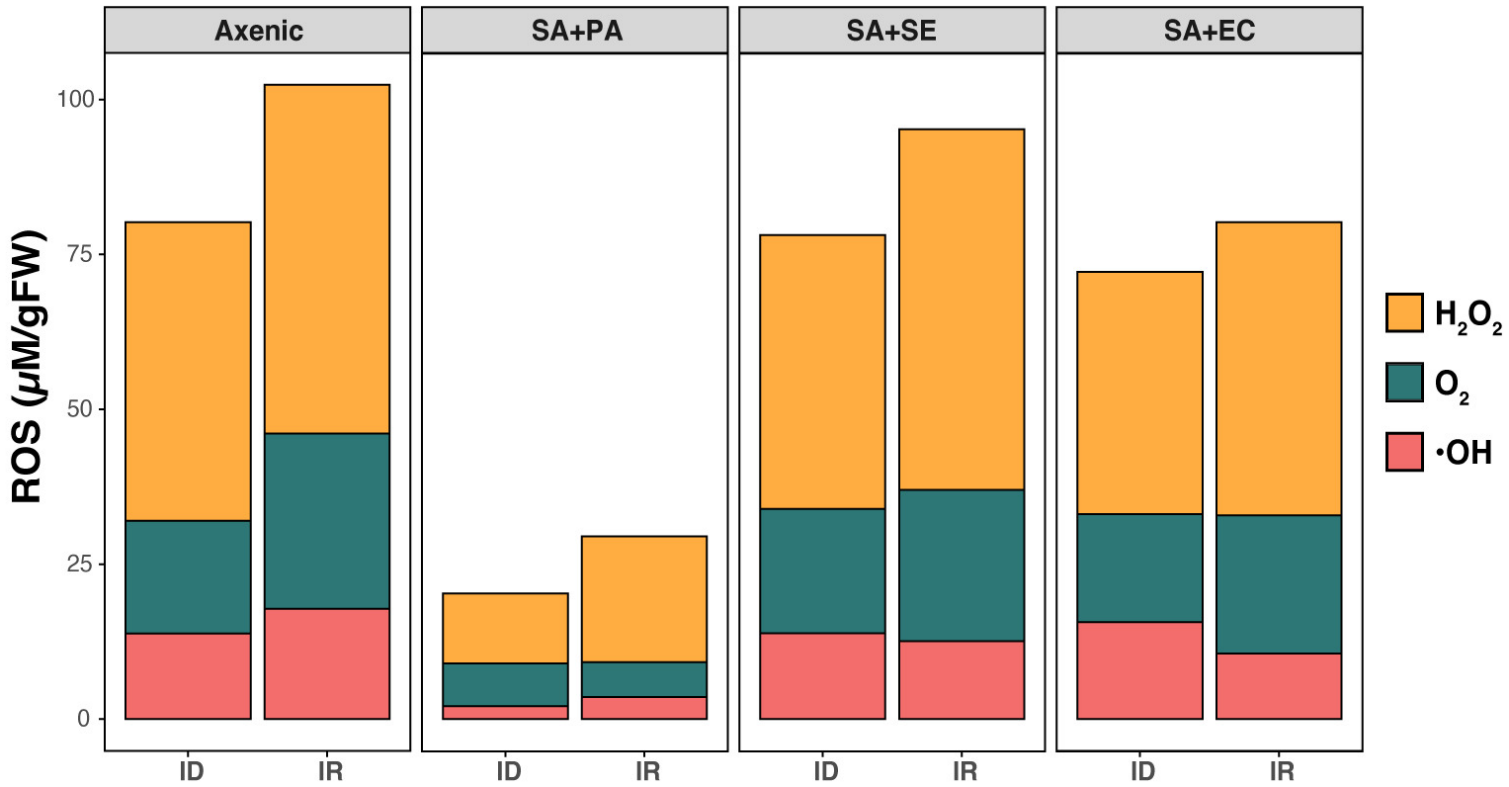
Comparative analysis of ROS production in axenic and co-cultured *S. aureus.* Production of different ROS molecules including H_2_O_2_, O_2_, and OH. were determined in both axenic and co-cultured *S. aureus* in both iron rich (IR) and iron deficient conditions (ID) at 48 h. Axenic: axenic *S. aureus*, SA+PA: Measurement of viable cells of *S. aureus* when co-cultured with *P. aeruginosa* in another compartment, SA+SE: Measurement of viable cells of *S. aureus* when co-cultured with *S. epidermidis* in another compartment, SA+EC: Measurement of viable cells of *S. aureus* when co-cultured with *E. coli* in another compartment.

In iron rich conditions, we observed the highest level of H_2_O_2_ (48.10 ± 4.12 µM/g FW) in control (axenic) group, while a subtle decrease was observed in the co-cultured SA with SE and EC. Interestingly, a much lower level (15.21 ± 3.08 µM/g FW) was found in co-cultured SA+PA (**Figure 3**). O_2_ and OH contents were not significantly different between the axenic and co-cultured strains. Moreover, an increase of oxidative stress was also observed in iron-deficient conditions, showing the highest H_2_O_2_ levels (57.10 ± 1.02 µM/g FW) in co-cultured SA+SE as well as other axenic and co-cultured strains. Although O_2_ production displayed a similar pattern in SA+SE and SA+EC cultures, it was comparatively lower than in the axenic cultures. However, with respective to iron content, it was higher in iron-rich media in all the strains except the co-cultured SA+PA, which showed the least amount (7.16 ± 0.41 µM/g FW). This can be correlated with the viability of cells, where, except for co-cultured SA+PA, other co-cultured bacteria showed a comparatively lower number of live cells and a decreased growth rate. From **Figure 3**, it is observed that under both iron-rich and iron-deficient conditions, ROS accumulation increased in all other bacterial cultures, except in SA+PA.

### 3.5 Phenotypic differentiation caused by siderophore dynamics

**Figure 4:**
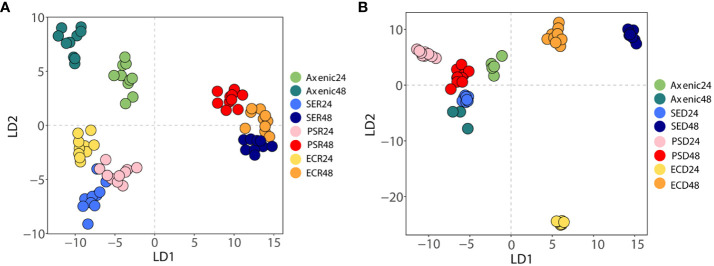
A discriminant analysis of principal components of Raman spectra of axenic and co-cultured *S. aureus* at different time intervals using Raman spectroscopy. **(A)** Phenotypic changes between axenic and co-cultured *S. aureus* at 24 and 48 h in iron-rich conditions. **(B)** Phenotypic changes between axenic and co-cultured *S. aureus* in 24 and 48 h in iron-deficient conditions. Different colors denote different bacterial species.

**Figure 5:**
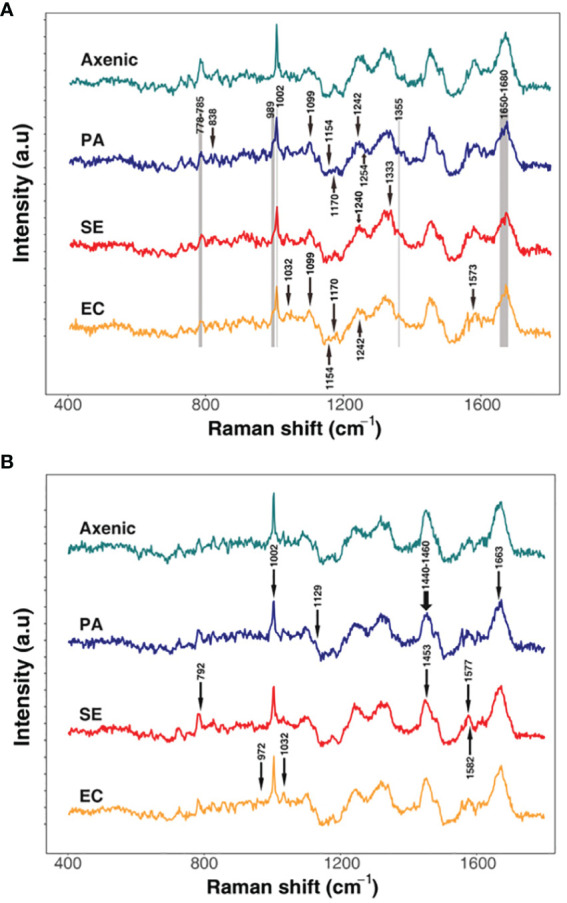
Differential Raman spectroscopic signatures of *S. aureus* in response to varied co-culture conditions in iron rich **(A)** and iron-deficient conditions **(B)**. The arrows represent unique peaks of the co-cultured SA that have specifically changed compared to the axenic culture. Grey areas indicate common peaks that have changed in all the samples compared to the axenic cultures.

Raman spectroscopy can be used to analyze the effects of interactions of microbial phenotypes at the molecular level (Cui et al., 2022). We collected Raman spectra representing the phenotype of SA that altered with iron levels and co-culturing with bacterial types and visualized by DAPC, as depicted in **Figure 4**. The Raman spectra obtained from co-culturing SA with the other three microbial species in an iron-rich environment were tightly clustered after 24 and 48 h (**Figure 4A**). Each cluster was significantly distant from the monoculture phenotype regardless of time. The negligible difference in the phenotype altered by co-culturing with other microorganisms at a given time-point indicates that, in iron-rich environments, the co-culture itself had a high impact on the SA phenotype. These results were confirmed by Raman spectra of the monoculture phenotype of SA with that obtained after co-culture, which showed that the Raman peaks or bands related to nucleic acids (778-785 cm^-1^ and 1355 cm^-1^), proteins (989 cm^-1^), amino acids (1002 cm^-1^), and lipids (1650-1680 cm^-1^) (**Figure 5A**; **Table 3**) commonly shifted in the co-cultured phenotype. In addition to common phenotypic changes, we observed Raman spectra that were specific to the species co-cultured with SA. Co-incubation with PA and EA changed the Raman peaks associated with proteins (838 cm^-1^ or 1170 cm^-1^) and amide (1242 cm^-1^, 1154 cm^-1^ or 1573 cm^-1^), while co-incubation with SE changed nucleic acids peaks (1240 cm^-1^ and 1333 cm^-1^).

**Table 3:** Assignment of Raman peaks or bands of *Staphylococcus aureus*.

Raman Wavenumber (cm^-1^)	Assignment	Group
778-785	Cytosine, Uracil (ring, str)	Cytosine, Uracil
792	Cytosine, Uracil (ring, str)	Cytosine, Uracil
838	DNA	DNA
972	CH2 rock., C–C stretch., α-helix	Proteins, lipids
989	β-sheet	Proteins, histamine
1002	Phenylalanine	Phenylalanine, b-carotene
1099	CC sKel, CoC a-str, PO2 str	
1032	Phenylalanine; C-N str	Phenylalanine
1129	ν(C−N)	Cyt c.
1154	C-C, C-O, C-N, r(CH3)	b-carotene
1170	C–H in-plane bend. mode (Tyr), (CH)	Proteins
1240	Thymine, cytosine, adenine, ring ν	Thymine, cytosine, adenine
1242	Amide III	Amide III
1254	Adenine, amide III	Adenine, amide III
1333	CH3CH2 def. of collagen	Nucleic acids, proteins
1355	A, G, CH def.	Nucleic acids, proteins
1440-1460	C-H2 def	
1453	Protein	Proteins
1573	C=C, N–H def, and C–N str (amide II)	amide II
1577	Guanine, Adenine ring stretching	Guanine, Adenine
1582	Protein	Proteins
1650-1680	Amide I	Amide I
1663	Amide I	Amide I

In iron-deficient environments, the phenotype of SA changed specifically depending on the species co-cultured (**Figure 5B**; **Table 3**). The phenotype of SA co-cultured with EC changed dramatically and was significantly distant from that of the other phenotypes after 24 h, whereas the phenotype of SA co-cultured with PA and SE was not significantly different from that of monocultured SA. The phenotype of SA co-cultured with EC and SE was uniquely distant from that of the monocultured SA after 48 h, whereas the phenotype of SA co-cultured with PA was very similar to that of monocultured SA. Unlike those under iron-rich conditions, we could not identify any commonly shifted Raman peaks or bands among the co-cultured phenotypes of SA under iron-deficient conditions (**Figure 5B**). Species-specific Raman spectra, such as those from iron-rich environments, also did not overlap across species. Co-culturing with PA altered the cytochrome (1129 cm^-1^) and amino acids (1002 cm^-1^) peaks of SA. Co-culture with SE changed nucleic acids (792 cm^-1^ and 1577 cm^-1^) and proteins (1453 cm^-1^) peaks, whereas co-culture with EA changed the proteins/lipids (972 cm^-1^) and amino acids (1032 cm^-1^) peaks. These results suggest that siderophore dynamics induced by microbial interactions strongly influenced the phenotypic differentiation of SA under iron-deficient conditions.

## 4 Discussion

In this study, we optimized culture media for production of diverse siderophore and analyzed the effects of co-cultivation on the dynamics of siderophore production in SA. We found that the GAST medium was the most suitable for studying siderophore production because it supports stable production of all three types of siderophores after 24 h. A combination of glycerol and alanine in the medium, which served as carbon and nitrogen sources, respectively, significantly influenced the production of various functional groups of siderophores in SA. Carbon sources play a crucial role in siderophore production (Santos et al., 2014; Musialowski et al., 2023). Moreover, the presence of the amino acid, alanine, in the GAST medium helped in the quick assimilation of glycerol (a complex molecule), that promoted the bacterial growth and also assisted in the production of various types of siderophores. Amino acids are also involved in boosting siderophores. Therefore, L-alanine may have acted as a building block for the production of staphyloferrin A, which contains an ornithine chain, and other siderophores, as described in previous studies (Sayyed et al., 2019; Dileep et al., 2021). Since this study relates to the co-cultivation of four different bacterial strains, selecting an iron-deficient medium that supports the growth and production of siderophores in all strains was challenging. The GAST medium was eventually selected to study the dynamics of siderophores, considering its support in the growth of all the selected bacterial strains and siderophore production in SA.

Our investigation of siderophore production under co-culture conditions revealed crucial insights that differed from those of traditional monoculture studies. Our study highlighted the critical functions of microbial interactions in the biosynthesis of these iron-scavenging molecules. In a competitive microbial environment (e.g., iron-rich conditions), diverse siderophore production patterns were observed in SA, which were mainly influenced by the co-cultivated microbes. These findings highlighted the adaptive strategies employed by this species in shared ecological niches. In co-culture with PA, siderophore production in SA ceased completely, plausibly to preserve resources in response to competitive pressures or because of the availability of hydroxamate siderophores produced by PA (Woods et al., 2019; Perraud et al., 2020). This result is consistent with previous findings, which reported that the presence PA along with SA provides SA with an opportunity to cheat trait through upregulation of genes involved in the production of siderophores in PA, thus increasing siderophore production (Yang et al., 2020). The benefit of SA in this situation is explained by the relatively low ROS and high viability of SA when co-cultured with PA compared to co-culturing with other species. In contrast, co-cultivation of SA with SE or EC resulted in an intriguing change in the siderophore profile. In the former, SA produced a combination of carboxylate and hydroxamate siderophores, whereas in the latter, significantly higher amounts of carboxylate siderophores were secreted. This indicates a collaborative or compensatory approach and highlights the complexities of microbial interactions (Carroll and Moore, 2018). Although, in this study, it is not clear as to why SA produced only a particular siderophore out of the three siderophores, we can surmise that this behavior is an attempt to gain a competitive advantage in the shared habitats with other microorganisms. Changes in siderophore production can have a substantial impact on microbial ecology, altering competitive dynamics within microbial communities. For instance, when cocultured with SA, certain strains may reduce or even stop producing siderophores, which could decrease their competitiveness for iron (Kramer et al., 2020). These findings indicate that interspecies interactions among microorganisms significantly affect siderophore diversity and production dynamics, understanding serves to deepen our ecological insights into the roles and functions of *Staphylococcus* within complex microbial communities. This approach to microbial ecology brings us one step closer to understanding the nuances of microbial survival and cooperative strategies in shared habitats.

Microbial interactions and siderophore dynamics have been found to play a crucial role in directing phenotypic differentiation of SA, especially under iron-deficient conditions. Our research utilized Raman spectroscopy to examine microbial phenotypes at the molecular level, which sets our study apart from previous studies and provides a more nuanced understanding of bacterial adaptations. Co-culturing influenced the phenotype of SA in iron-rich environments regardless of the specific species with which it was paired. This suggests that the process of co-culturing has a significant impact on the phenotype of SA. Observing the shifts in Raman peaks or bands related to nucleic acids, proteins, amino acids, and lipids, directed our attention to the broader context of microbial interactions in shaping bacterial phenotypes, beyond species specificity (Cialla-May et al., 2021). Under iron-deficient conditions, the phenotypic differentiation of SA was influenced by the species with which it was co-cultured. This highlights the importance of interspecies interactions in bacterial adaptation to environmental stressors (Scheuerl et al., 2020). An example of the significant impact of microbial interactions on the phenotypic differentiation of SA could be seen in its distinctly different phenotype when co-cultured with EC, whereas no considerable changes were observed when co-cultured with PA. Microorganisms effectively adapt to low-resource environments, particularly under conditions of low iron, by producing siderophores that outcompete other microbes for the scarce available iron resource. In the co-cultivation with EC, peaks associated with proteins/lipids at 972 cm^-1^ and amino acids at 1032 cm^-1^ showed significant shifts. The alteration in lipids/proteins peaks could represent membrane remodeling, possibly as a response to alterations in siderophore export or import mechanisms (Pezzotti, 2021). Amino acids serve as the building blocks for siderophores, and changes in their Raman peaks may indicate a shift in the metabolic flux towards different siderophore types. In the co-cultivation of SA with SE, notable variations were detected in the Raman peaks corresponding to nucleic acids at 792 cm^−1^ and 1577 cm^−1^, as well as for proteins at 1453 cm^−1^. Given that nucleic acids act as indicators for genomic regulatory changes, these spectral shifts in nucleic acid peaks could symbolize modifications in the gene expression profiles related to siderophore production or trafficking (Pezzotti, 2021). Similarly, the observed alterations in protein-associated peaks may reflect a transformation in the enzymatic milieu governing the biosynthesis of siderophores. Microorganisms can also utilize siderophores produced by other bacteria (known as ‘siderophore piracy’) depending on the presence of other species. This allows them to maximize their fitness without investing heavily in their own siderophore production (Andrić et al., 2023). This dual strategy related to production and piracy allows microorganisms to become competitive and flexible in response to different species and environmental conditions. Furthermore, these phenotypic changes and alterations in siderophore production may have significant implications for host-pathogen interactions. Different SA phenotypes may display different degrees of virulence, resistance to antibiotics, and capacity to evade host immune responses (Howden et al., 2023). The ability of SA to modulate its production of siderophores in response to the presence of other bacterial species may also affect its pathogenicity, conceivably influencing the course of diseases and results of treatment (Chadha et al., 2022).

## 5 Conclusion

The present study has significantly improved our understanding of siderophore dynamics and phenotypic differentiation in *S. aureus*, particularly under iron-deficient conditions. We successfully optimized the GAST medium, which enables diverse production of siderophores by *S. aureus* under co-cultivation conditions. Our results indicate that microbial interactions substantially influence the production of siderophores, thereby affecting microbial ecology and competitive dynamics within microbial communities. Phenotypic differentiation of *S. aureus* was substantially influenced by the specific species with which it was co-cultured, indicating the crucial role of interspecies interactions in bacterial adaptation to stressful conditions. Overall, this study not only deepens the understanding of microbial ecology and strategies for microbial survival but also identifies the role of *S. aureus* within complex microbial communities in iron-deficient conditions. Thus, our research marks a significant step forward in understanding microbial interactions and their effects on siderophore production and phenotypic differentiation.

## Data availability statement

The original contributions presented in the study are included in the article/**Supplementary Material**. Further inquiries can be directed to the corresponding author.

## Author contributions

SR: Data curation, Investigation, Visualization, Writing – review & editing, Formal Analysis, Methodology, Software, Writing – original draft. EL: Data curation, Methodology, Software, Visualization, Writing – review & editing. BK: Data curation, Software, Writing – review & editing, Visualization. TL: Data curation, Visualization, Writing – review & editing, Conceptualization, Funding acquisition, Investigation, Resources, Supervision, Validation.
