# Supplementary material for: Microbial interaction-induced siderophore dynamics lead to phenotypic differentiation of Staphylococcus aureus
Source: Front Cell Infect Microbiol. 2023 Nov 17;13:1277176. doi: 10.3389/fcimb.2023.1277176 (PMC10690949; doi:10.3389/fcimb.2023.1277176)
Supplement: Supplementary file 1 [file DataSheet_1.docx]

Supplementary Material

**Microbial interaction-induced siderophore dynamics lead to phenotypic differentiation of *Staphylococcus aureus***

Soundarya Rajapitamahuni **^1^,** Eun Sun Lyou **^1^,** Bo Ram Kang **^1^,** and Tae Kwon Lee **^1 *^**

**^1^** Department of Environmental and Energy Engineering, Yonsei University, Wonju 26493, Republic of Korea

*Correspondence

Tae Kwon Lee

Email: tklee@yonsei.ac.kr; Tel: +82-33-760-2446; Fax: +82-33-760-5524

**Keywords**: Siderophores, Microbial interactions, Iron deficiency, Phenotype, Raman Spectroscopy

**Supplementary Figure captions**

**Supplementary Figure 1**. Growth rate of *S. aureus* in both axenic and co-culture conditions in iron rich and iron deficient conditions at (optical density OD measured at 600nm) at 48 h. X axis denotes axenic *S. aureus* (When *S. aureus* is present in both compartments) and *S. aureus* co-cultured/ grown in transwell plates where in, one compartment contains *S. aureus* and other comparment contains other species. Axenic: axenic *S. aureus*, SA+PA: Measurement of viable cells of *S. aureus* when co-cultured with *P. aeruginosa* in another compartment, SA+SE: Measurement of viable cells of *S. aureus* when co-cultured with *S. epidermidis* in another compartment, SA+EC: Measurement of viable cells of *S. aureus* when co-cultured with *E. coli* in another compartment

**Supplementary Figure 2**. Determining Live %(Blue) and Dead cells %(Red) of axenic and co-culture *S. aureus* through flow cytometry in both iron rich and deficient conditions at 48 h. X axis denotes axenic *S. aureus* (When *S. aureus* is present in both compartments) and *S. aureus* co-cultured/ grown in transwell plates where in, one compartment contains *S. aureus* and other compartment contains other species. Axenic: axenic *S. aureus*, SA+PA: Measurement of viable cells of *S. aureus* when co-cultured with *P. aeruginosa* in another compartment, SA+SE: Measurement of viable cells of *S. aureus* when co-cultured with *S. epidermidis* in another compartment, SA+EC: Measurement of viable cells of *S. aureus* when co-cultured with *E. coli* in another compartment

**Supplementary Figure 3**. Growth rate of co-cultures of other three bacteria (optical density OD measured at 600nm) at 48 h. **(A)** Difference in axenic and effect of co-cultivation on *P. aeruginosa* (PA), *S. epidermidis* (SE), *E. coli* (EC) in Iron rich conditions **(B)** Difference in axenic and effect of co-cultivation on *P. aeruginosa* (PA), *S. epidermidis* (SE), *E. coli* (EC) in Iron deficient conditions. The data represents mean values of three replicates with the error bars indicating their standard deviations.


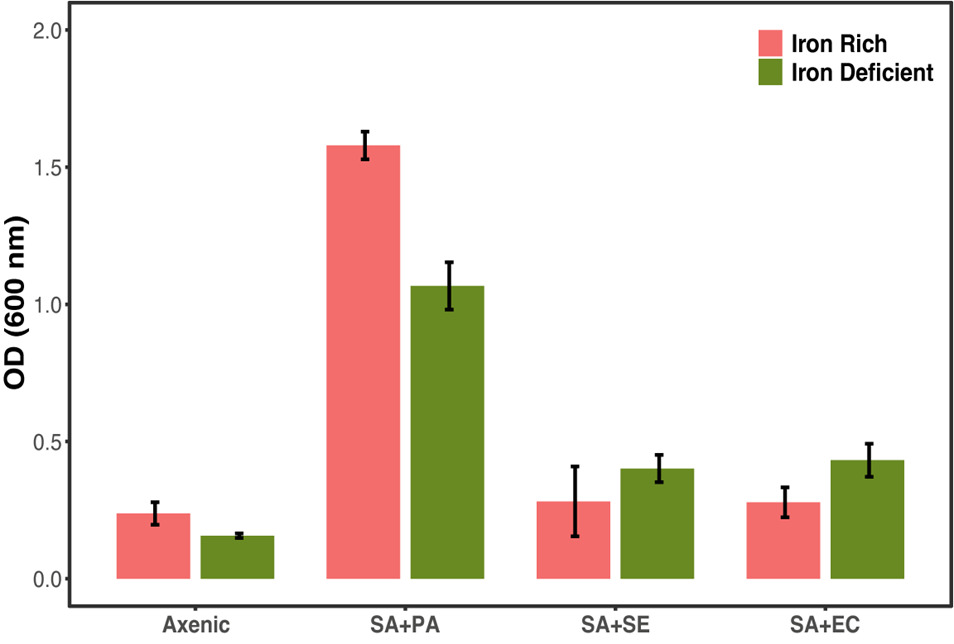


**Supplementary Figure 1**. Growth rate of *S. aureus* in both axenic and co-culture conditions in iron rich and iron deficient conditions at (optical density OD measured at 600nm) at 48 h. X axis denotes axenic *S. aureus* (When *S. aureus* is present in both compartments) and *S. aureus* co-cultured/ grown in transwell plates where in, one compartment contains *S. aureus* and other compartment contains other species. Axenic: axenic *S. aureus*, SA+PA: Measurement of viable cells of *S. aureus* when co-cultured with *P. aeruginosa* in another compartment, SA+SE: Measurement of viable cells of *S. aureus* when co-cultured with *S. epidermidis* in another compartment, SA+EC: Measurement of viable cells of *S. aureus* when co-cultured with *E. coli* in another compartment


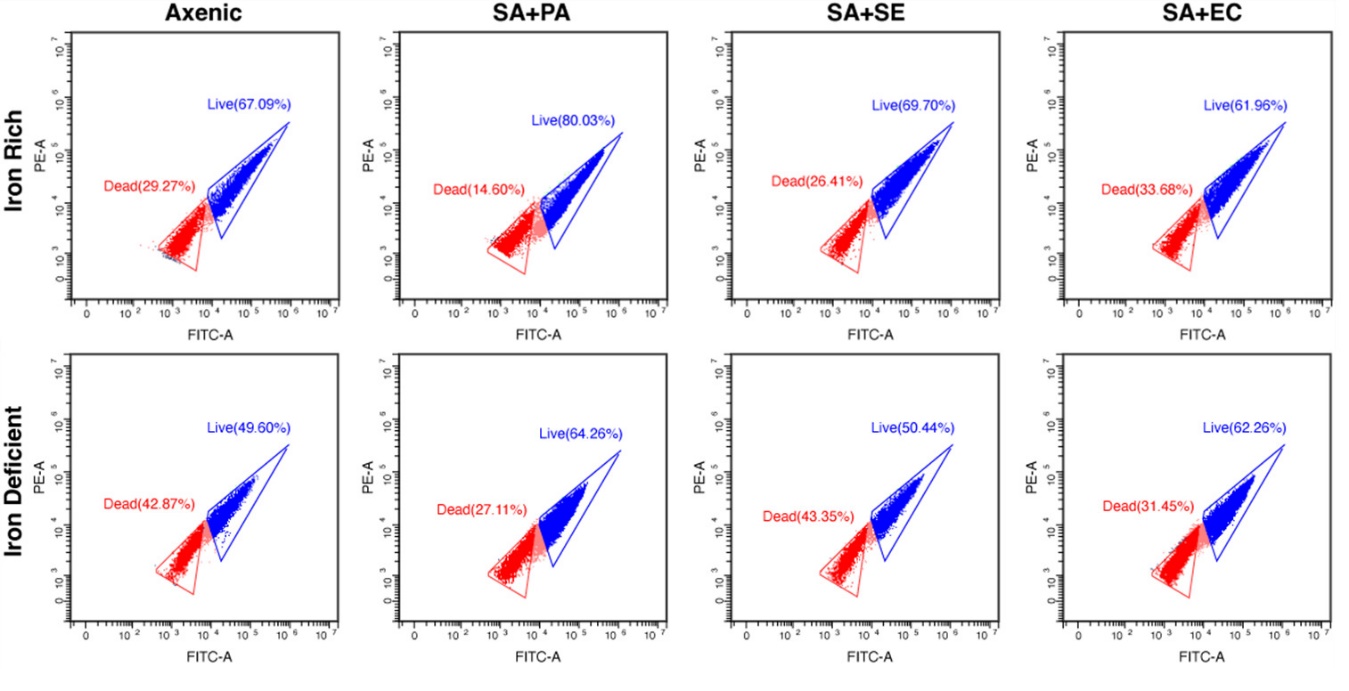


**Supplementary Figure 2**. Determining Live %(Blue) and Dead cells %(Red) of axenic and co-culture *S. aureus* through flow cytometry in both iron rich and deficient conditions at 48 h. X axis denotes axenic *S. aureus* (When *S. aureus* is present in both compartments) and *S. aureus* co-cultured/ grown in transwell plates where in, one compartment contains *S. aureus* and other compartment contains other species. Axenic: axenic *S. aureus*, SA+PA: Measurement of viable cells of *S. aureus* when co-cultured with *P. aeruginosa* in another compartment, SA+SE: Measurement of viable cells of *S. aureus* when co-cultured with *S. epidermidis* in another compartment, SA+EC: Measurement of viable cells of *S. aureus* when co-cultured with *E. coli* in another compartment


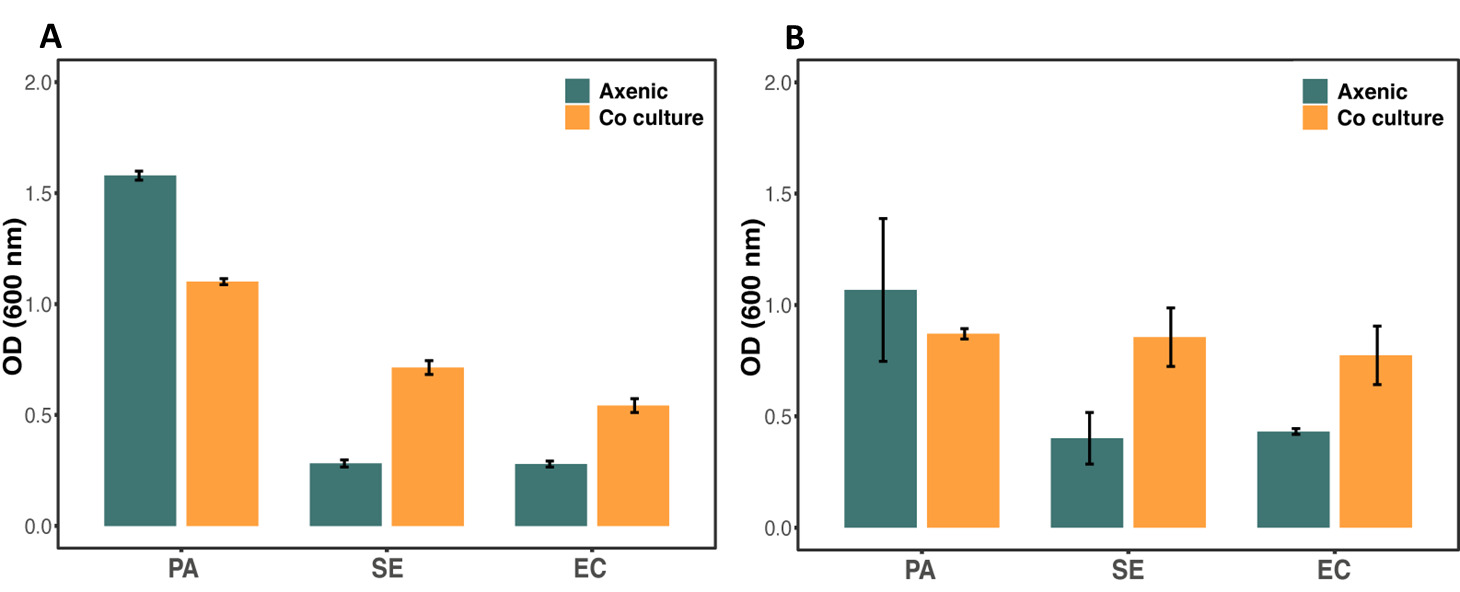


**Supplementary Figure 3**. Cell density of co-cultures of other three bacteria (optical density OD measured at 600nm) at 48 h. **(A)** Difference in axenic and effect of co-cultivation on *P. aeruginosa* (PA), *S. epidermidis* (SE), *E. coli* (EC) in Iron rich conditions **(B)** Difference in axenic and effect of co-cultivation on *P. aeruginosa* (PA), *S. epidermidis* (SE), *E. coli* (EC) in Iron deficient conditions. The data represents mean values of three replicates with the error bars indicating their standard deviations.
